# Navigating Road Traffic Accidents in Jordan: A Retrospective Exploration of the Health and Economic Impacts

**DOI:** 10.7759/cureus.60497

**Published:** 2024-05-17

**Authors:** Ala'a B Al-Tammemi, Shahd A Altarawneh, Linda Zaid Abu Ghazaleh, Tala M Jaradat, Raghad Abujudeh, Murad Almbaidin, Hanan Hasan

**Affiliations:** 1 Research, Policy, and Training Directorate, Jordan Center for Disease Control (JCDC), Amman, JOR; 2 Faculty of Nursing, Jordan University of Science and Technology, Irbid, JOR; 3 Department of Civil Engineering, Jordan University of Science and Technology, Irbid, JOR; 4 Department of Basic Medical Sciences, Faculty of Medicine, Jordan University of Science and Technology, Irbid, JOR; 5 Division of Public Health, Naour Comprehensive Health Center, Jordanian Ministry of Health, Amman, JOR; 6 Migration Health Division, International Organization for Migration (IOM), Amman, JOR; 7 Quality and Patient Safety, Al-Waleed Medical Laboratories, Amman, JOR

**Keywords:** traffic awareness, economic burden, health burden, traffic violations, road safety, road traffic injuries, road traffic accidents, jordan

## Abstract

Background

Road traffic accidents (RTAs) are considered a major public health threat. The causes of RTAs are multifactorial, comprising both human and non-human factors. RTAs may result not only in detrimental health consequences but also in serious economic burdens. This study aimed to provide a retrospective overview of the health and economic impacts of RTAs in Jordan during the period 2013-2021, including the implemented multisectoral mitigation strategies.

Methodology

This study presents a retrospective chart review of the traffic reports published by the Public Security Directorate (PSD) in Jordan during the period 2013-2021. The examined variables were the total number of road accidents involving human injuries per year, average road accidents per day, annual deaths, average deaths per day, deaths per 100,000 population, annual number of injuries, average number of injuries per day, severity of human injury, number of registered vehicles, and the annual financial costs. Data were extracted from PSD reports and presented descriptively.

Results

The number of registered vehicles has risen from 1,263,754 in 2013 to 1,795,215 in 2021 with a 42.1% increase. This was also associated with an increase in the average RTAs per day from 28.0 in 2013 to 30.8 in 2021. The total number of RTAs that involved human injuries during the period 2013-2021 fluctuated with 8,451 in 2020 (lowest) and 11,241 in 2021 (highest). In the same period, annual deaths caused by RTAs peaked in 2013 with 768 deaths (highest) compared to 461 in 2020 (lowest). In the five years from 2017 to 2021, the total financial cost of RTAs in Jordan was nearly 1.561 billion Jordanian Dinars (~2.2 billion U.S. dollars) compared to 1.363 billion Jordanian Dinars (~1.92 billion U.S. dollars) in the five years from 2012 to 2016.

Conclusions

Targeting human and road infrastructure factors through effective government-society partnerships is crucial in mitigating the health and economic burden of RTAs in Jordan. More strict enforcement of traffic laws, along with incremental increases in the penalties for dangerous traffic violations, and regular road safety campaigns are expected to provide more control over the human factors of RTAs. Further research is needed to evaluate the impact of the recently amended traffic law on the trends of RTAs in the country.

## Introduction

Road traffic accidents (RTAs) are considered a serious and notable public health threat worldwide, with 93% of crash fatalities occurring in low and middle-income countries according to a recent report by the Global Road Safety Facility [1]. Worldwide, the incidence of road injuries has increased from 63.2 million in 1990 to 103.2 million in 2019 [2]. This was also accompanied by an increase in road injuries-related deaths from 1.11 million in 1990 to nearly 1.2 million in 2019 [2]. Furthermore, RTAs attract plenty of public attention due to the negative consequences on road safety among drivers and pedestrians, as well as the associated fatalities and injuries [3]. Globally, more than 1.3 million deaths and 50 million injuries and disabilities are attributed to road injuries annually [2]. RTAs are considered the leading cause of death among children and young adults in the age group of 5-29 years [4,5]. Additionally, RTAs are associated with serious economic burdens in terms of direct and indirect financial losses, such as costs of hospitalizations and treatment, long-term rehabilitation expenses, property loss, productivity loss, legal costs, and insurance costs [2,5].

There are many reasons and risk factors that increase the likelihood of RTAs and road injuries. Human factors such as speeding, aggressive driving, and road rage; driving under the influence of alcohol or psychoactive substances; non-use of seatbelts, child restraints, and motorcycle helmets; and distracted driving while using mobile phones are considered major risk factors for RTAs [4-6]. In addition, unsafe road infrastructure and design, unsafe vehicles, and insufficient traffic law enforcement are considered major contributing factors to RTAs [3,4,6,7]. Moreover, hazardous weather conditions such as dust, ice, snow, and heavy rains contribute to the occurrence of RTAs, especially if safety measures are neglected by the drivers. Recent literature reported that more than 90% of RTAs are caused by human errors [8,9].

Jordan is a middle-income country located in the World Health Organization (WHO) Eastern Mediterranean Region (EMR) with a population of nearly 11.5 million [10-12]. Jordan occupies a strategic location in the EMR and shares land borders with many countries, including Iraq, Palestine, Saudi Arabia, and Syria. The country consists of 12 governorates distributed over three geographical regions (Northern, Central, and Southern regions) [13,14]. Nevertheless, the country faces significant challenges that are shaped in many pillars, most importantly, its location in a politically and economically unstable region, as well as being a country with limited resources. These challenges impose a serious burden on various vital sectors of the country, including transport, health, finance, manufacturing, and tourism [15].

According to statistics revealed by the Jordan Investment Commission and the Economic Policy Council, the transportation sector is considered one of the main sectors in Jordan with around 9.0% of contribution to Jordan’s gross domestic product [15,16]. During the past decades, and in parallel with the notable growth in population and vehicles, Jordan has heavily invested in improving the transport sector through various pillars to meet the demands of the growth. This included capacity building and road expansion, enhancing road infrastructure, improving urban transport, and opening the transport market to private operators [17]. The length of the road network in Jordan is approximately 8,000 km with around 3,400 km classified as main roads [14,18]. Currently, the Jordanian population is highly dependent on cars for personal mobility, which is also influenced by how people drive (behavior of drivers). Despite the presence of many traffic laws and legislations that aim at reducing traffic hazards and enhancing road capacity, traffic violations, especially fatal ones, are increasingly alarming in Jordan [17,19].

This study aimed to provide a descriptive and retrospective overview of the health and economic impacts of RTAs in Jordan during the period 2013-2021, including the implemented multisectoral mitigation strategies.

## Materials and methods

A retrospective chart review was conducted for the publicly available annual traffic reports published by the Public Security Directorate (PSD) in Jordan during the period 2013-2021 [19]. The annual traffic reports provide a comprehensive analysis of data to assess trends, patterns, and key indicators related to RTAs, injuries, and fatalities in Jordan. To facilitate this retrospective examination, the research team defined the research aims, and then the sources of data were determined. The next step was selecting the needed variables, followed by extracting relevant data from the traffic reports. Multiple authors were engaged in reviewing the annual reports to accurately extract the required data. The research team meticulously reviewed the traffic reports and extracted relevant variables that needed to be examined in the current study. These variables included the annual number of road accidents involving human injuries, average road accidents per day, annual deaths due to road accidents, average deaths per day, deaths per 100,000 population, total number of injuries per year, average number of injuries per day, severity of human injury (mild-to-moderate versus severe), number of registered vehicles, and annual financial costs. Additionally, the authors reviewed the multisectoral efforts and initiatives that aim to combat RTAs in the country, as well as Jordan’s Road Safety Strategy 2019-2023, including its main pillars and objectives.

We present here a descriptive analysis of the above-mentioned variables utilizing tables and graphical displays. As the PSD’s annual traffic reports are publicly available to users and researchers, and due to the non-involvement of human subjects in this study, obtaining ethical approval was not required to conduct this retrospective chart review.

## Results

The number of registered vehicles in Jordan expanded from 1,263,754 in 2013 to 1,795,215 in 2021 with a 42.1% increase [19]. This was also associated with an increase in the average RTAs per day from 28.0 in 2013 to 30.8 in 2021. Table 1 presents more details about the statistics of RTAs in Jordan during the period 2013-2021.

**Table 1:** Annual statistics of road traffic accidents (RTAs) in Jordan during 2013-2021. Data were extracted from the Public Security Directorate’s annual traffic reports.

	2013	2014	2015	2016	2017	2018	2019	2020	2021
Average number of road accidents per day	28.0	26.7	26.6	29.7	28.6	28.6	29.7	23.2	30.8
Average deaths per day due to road accidents	2.1	1.9	1.7	2.1	1.9	1.6	1.8	1.3	1.6
Road accident-related deaths per 100,000 population	11.76	10.31	6.38	7.65	6.81	5.5	6.1	4.3	5.3
Average number of injuries per day	43.7	40.5	44.2	47.8	44.5	44.4	46.6	34.8	47.9
Number of registered vehicles	1,263,754	1,331,563	1,412,817	1,502,420	1,583,458	1,637,981	1,677,061	1,729,343	1,795,215

**Figure 1:**
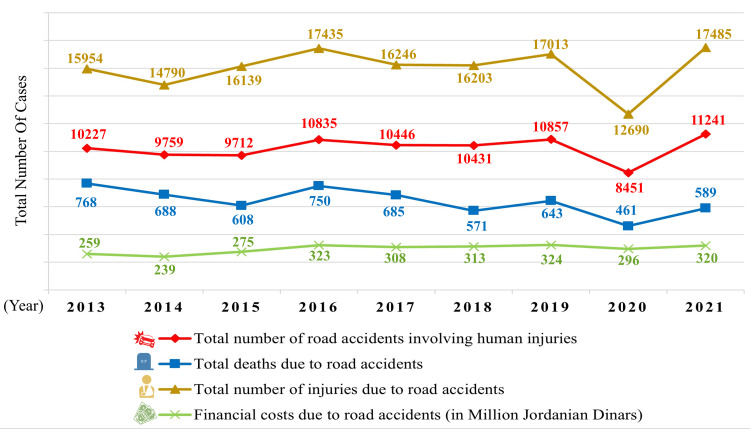
Trends of road traffic accidents (RTAs) in Jordan and the related financial costs during the period 2013-2021. One Jordanian Dinar = 1.41 U.S. dollars. The financial cost in 2012 was 267 million Jordanian Dinars. Data were extracted from the Public Security Directorate’s annual traffic reports.

**Figure 2:**
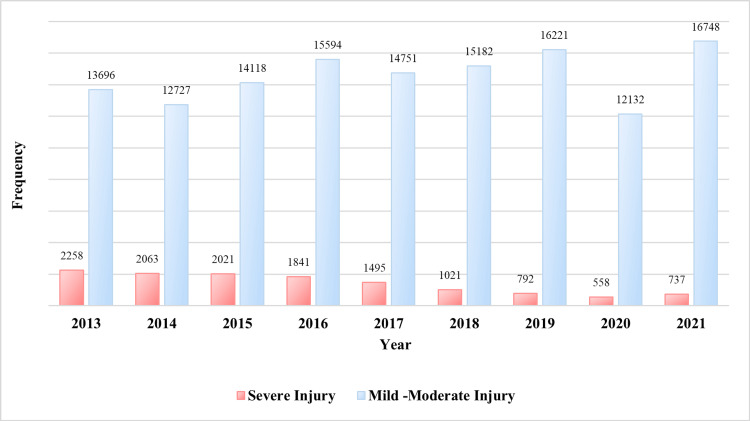
Severity classification of road traffic injuries during the period 2013-2021. Data were extracted from the Public Security Directorate’s annual traffic reports.

As per annual reports from the Jordanian PSD, the total number of RTAs that involved human injuries during the period 2013-2021 fluctuated with 8,451 in 2020 (lowest) and 11,241 in 2021 (highest), as shown in Figure 1. In the same period, yearly deaths caused by RTAs peaked in 2013 with 768 deaths (11.76 per 100,000 population) compared to 461 in 2020 (4.3 per 100,000 population), as shown in Figure 1 and Table 1. Additionally, Figure 2 demonstrates the severity of human injuries per year.

In the five years from 2017 to 2021, the total financial cost of RTAs in Jordan was nearly 1.561 billion Jordanian Dinars (~2.2 billion U.S. dollars) compared to 1.363 billion Jordanian Dinars (~1.92 billion U.S. dollars) in the five years from 2012 to 2016, noting that the financial cost in 2012 was 267 million Jordanian Dinars (not presented in the figure) (see Figure 1).

## Discussion

To our knowledge, this is the first study to provide an overview of the health and economic consequences of RTAs in Jordan during the period 2013-2021, utilizing data from PSD’s annual traffic reports. According to our findings, the average RTA deaths per day were the highest in 2013 and 2016 with 2.1 deaths per day, while 2020 registered the lowest rate with 1.3 deaths per day. The reason for such a low RTA-related death rate per day in 2020 could be attributed to the country’s lockdown and nationwide curfew which were forced by the Jordanian government as a mitigation measure against the COVID-19 pandemic [12,20]. This was also noticed in the European region where RTA deaths fell by 13% in 2020 due to the COVID-19 pandemic measures [21]. Consequently, and due to the stringent measures that restricted people’s mobility during the curfews and the associated minimal usage of vehicles, the number of RTAs that involved human injuries in 2020 was found to be 8,451, which is the lowest in the examined period (2013-2021) [19]. In 2022, a press release revealed the occurrence of around 170,000 road accidents in Jordan (with and without human injuries), resulting in 562 deaths in the same year [22].

Other countries in the region are also impacted by the health and economic consequences of RTAs. In 2017, there were 185,251 traffic-related deaths in the EMR. In 2017, age-standardized death rates related to RTAs in the EMR were the highest in Somalia, UAE, Oman, Yemen, and Afghanistan, and the lowest were in Jordan, Iraq, Bahrain, Palestine, and Lebanon. Compared to 1990, the EMR has witnessed a substantial decrease in traffic-related deaths and disability-adjusted life years by 30.5% and 34.4%, respectively [23]. It is challenging to compare countries due to the variation in the socioeconomic profiles and the road infrastructure between them. For instance, the developments in the EMR regarding road infrastructure and urban design may not necessarily lead to lowering traffic-related deaths and injuries [23]. This partially explains why some high-income countries in the EMR still experience high incidences of RTAs.

Various national multisectoral efforts are being implemented to reduce the health and economic burdens of RTAs in Jordan, accompanied by raising public awareness of safe driving habits. Nevertheless, road tragedies are still occurring despite all efforts. For instance, while celebrating World Traffic Day on May 04, 2023, Jordan recorded multiple devastating RTAs that resulted in seven deaths [24]. This was followed by more fatal RTAs that killed more than 12 persons in less than three hours on May 10, 2023 [25], a few days before the Seventh UN Global Road Safety Week which was celebrated on May 15-21, 2023. These statistical figures show the grim picture of fatal road accidents in Jordan.

As previously mentioned, RTAs are not only associated with physical injuries and disabilities but also with financial burdens and losses. In Jordan, the economic costs resulting from RTAs are alarming, considering the limited resources of the country. In the 10 years from 2012 to 2021, the total financial cost of RTAs in Jordan was approximately 2.93 billion Jordanian Dinars (~4.12 billion U.S. dollars). These costs, whether direct or indirect, add to the economic burden of the country.

Nationwide, more effective and efficient implementation of the Jordanian traffic laws is needed, taking into consideration the road accidents and injuries that occur daily in the country. As some of the previously imposed traffic violation penalties were insufficient to deter drivers from committing fatal traffic violations that cause serious accidents, and in immense efforts to mitigate RTA hazards in Jordan, an amended Jordanian traffic law was introduced in September 2023 [26].

The amended traffic law has shown more stringent measures to combat fatal traffic violations. For instance, fines for using mobile phones while driving have been raised from 15 Jordanian Dinars (21.2 U.S. dollars) to 50 Jordanian Dinars (70.5 U.S. dollars). Additionally, running a red traffic light may result in imprisonment and a fine of 200-300 Jordanian Dinars (282-423 U.S. dollars). Furthermore, the amended law has increased the fines and penalties for other traffic violations such as crossing speed limits, processions, and not using seatbelts [26]. Doubling fines in case of repetition of the same fatal violations in the same year was also introduced, along with enforcing the traffic points system that collects traffic violation points yearly, which may result in the suspension of driving license if the violation points exceed 16 along with other disciplinary measures [26,27]. In addition, drivers who accumulate 8-15 penalty points must attend a specialized one-day training course at Jordan Traffic Institute (JTI), during which 8 points are deducted from their penalty record upon course completion [28].

Recognizing the urgency of addressing road accidents and injuries, Jordan has embarked on a comprehensive multisectoral approach aimed at mitigating the incidence and adverse effects of RTAs. By leveraging the expertise and resources of multiple sectors and stakeholders, Jordan is implementing a comprehensive road safety strategy that tackles the root causes of RTAs and improves the safety of the country’s roadways. These efforts were compiled through the road safety strategic plan 2019-2023 which outlines the collaborative multifaceted efforts among various ministries and sectors to enhance road safety and reduce traffic-related fatalities and injuries [29]. The pillars of this strategy and their corresponding objectives are presented in Table 2. Each area of focus involves specific objectives, actions, and responsibilities assigned to various governmental authorities, demonstrating the multifaceted approach that is being implemented in Jordan to mitigate road injuries and fatalities.

**Table 2:** Jordan’s Road Safety Strategy 2019-2023, including its pillars and the corresponding objectives.

Strategy pillar	Objectives
Regulatory Legislation, Enforcement, and Monitoring	Legislative enhancement to deter traffic violations and improve road safety. Strict monitoring of drivers’ behaviors and driving habits
Road Infrastructure Development	Evaluating and improving the road network to ensure road safety. Ensuring the safety of pedestrians, people with special needs, and the elderly. Monitoring the implementation of road infrastructure designs and standards. Integration of urban planning, land use, and transportation planning. Improving public transportation to reduce the private use of vehicles
Vehicles Safety	Improving and monitoring the mechanical readiness of vehicles, and ensuring the availability of public safety means in imported vehicles.
Traffic Research, Implementation Data, and Analysis	Providing information and data on road safety. Strengthening the role of scientific research, and supporting research activities related to traffic safety
Emergency Preparedness and Response	Promoting effective emergency response following an accident. Providing the ideal medical service for injured people in emergency centers after an accident
Public Awareness, Education, and Training	Raising traffic awareness and culture in the community and among road users. Ensuring well-trained drivers who are familiar with road dangers and first aid support
Partnerships and Management	Effective management and enhanced coordination/collaboration to improve road safety

Additionally, JTI, through joint efforts with civil and official authorities, is actively engaged in raising public awareness and capacity building of drivers and the community in general. These efforts include training courses, regular community workshops, nationwide traffic awareness campaigns, institutional lectures, social media campaigns, and an electronic traffic learning platform [28]. Various media channels are being actively used to raise public awareness of road safety through social media, broadcasting radio interviews with relevant authorities in collaboration with local radio stations, and distributing printed materials [28]. Among these efforts too, JTI is targeting school students by participating in the annual “Back to School” campaign to ensure students’ safety and safe arrival at their schools and houses as well as raising road safety awareness among this age group [28]. Collectively, these efforts are considered a cornerstone of improving driving habits and reducing the health and economic burden of RTAs in Jordan.

As the world is approaching the Eighth UN Global Road Safety Week, more efforts should be invested to raise public awareness about safe driving habits among drivers in Jordan. As more than 90% of road accidents are attributed to human errors, the Jordanian population has a significant responsibility and role in adopting safe driving habits and ensuring regular maintenance of vehicles. This can be achieved at the community level by raising public awareness regarding the catastrophic consequences of RTAs. In addition, large-scale awareness campaigns that are based on the constructs of the Health Belief Model (HBM) [30] are expected to have positive impacts on people’s driving behaviors. Drivers who adopt the constructs of the HBM and apply them to the issue of road injuries (perceived susceptibility, perceived severity, perceived benefits, perceived barriers, cues to action, and self-efficacy) are expected to practice safer driving habits which will help in preventing road accidents and injuries.

Furthermore, more sustainable efforts can be invested at the national level by implementing the global plan of action for road safety 2021-2030 which was developed by the WHO and the United Nations Regional Commissions, and this is considered a cornerstone for reducing the burden of RTAs in Jordan. This global plan encourages various governments and stakeholders to implement an integrated safe system approach to achieve safe roads. The main goal of this plan is to reduce the number of RTAs and the associated deaths and injuries by 50% during the period 2021-2030. According to the proposed global plan, this can be achieved through many pillars that involve multimodal transport and land-use planning, safe road infrastructure, vehicle safety, safe road use, and effective post-crash response.

Although using the existing PSD data is a strength as it leverages already collected data which is cost-effective and efficient for longitudinal analysis, our study has limitations that should be considered while interpreting the findings, some of which are not examining the dynamics of RTAs in Jordan in terms of timing of road accidents (days, weeks, and months), weather characteristics during which accidents occurred, vehicle speed at time of accidents, and the characteristics of victims (driver vs. pedestrian). Additionally, our study relies solely on secondary data which might not capture all relevant factors influencing RTAs, such as behavioral or psychosocial factors of drivers. The accuracy of the findings is contingent on the precision and comprehensiveness of the traffic reports. Any underreporting or inconsistencies in the data collection methods over the years would directly affect the study’s findings. As a descriptive study, it lacks a comparative framework that could potentially provide deeper insights, for example, comparing regions within Jordan that may have different traffic policies or infrastructure. Nevertheless, we aimed to provide a snapshot of the overall health and economic impacts, and we encourage further research on this topic considering the above-mentioned limitations.

## Conclusions

The causes of RTAs are multifactorial, and targeting human factors and road infrastructure factors properly through effective government-society partnerships has paramount importance in mitigating the burden of RTAs in Jordan. Working in solidarity to combat road injuries and reduce the associated health and economic burden in the country is pivotal.

Additionally, more strict enforcement of traffic laws, along with incremental increases in the penalties for dangerous traffic violations, and regular road awareness campaigns are expected to provide more control over the human factors of RTAs. This can be also supported by allocating sufficient funds for the maintenance and improvement of road infrastructure, encouraging sustainable transportation, and fostering the multisectoral and multifaceted collaboration between various stakeholders to address road safety comprehensively. From research and policy evaluation perspectives, further research is needed to evaluate the impact of the recently implemented Jordanian traffic law on the trends of RTA incidence and the associated morbidity, mortality, and financial burden. This could include modeling studies of RTAs to forecast the impact of amending a specific law or policy. Integrating hospital records or insurance claims to cross-validate the severity and financial estimates related to RTAs could enhance the robustness of future studies. Moreover, considering qualitative research could provide insights into the human factors causing RTAs, which are often not completely captured through quantitative data.
